# Daytime vs. Nighttime Surgery for Pediatric Supracondylar Humerus Fractures: A Retrospective Cohort Study

**DOI:** 10.3390/jcm15093282

**Published:** 2026-04-25

**Authors:** Alba Marrero Hernández, Maryé Mercé Méndez Ojeda, Eduardo Morales Pérez, Paula Couñago Parada, Nuria Álvarez Benito, Francisco Jesús Márquez Marfil

**Affiliations:** Department of Orthopaedic Surgery and Traumatology, Hospital Universitario de Canarias, 38320 Tenerife, Spain; alba199765@gmail.com (A.M.H.);

**Keywords:** pediatric supracondylar fracture, pediatric orthopaedic trauma, timing of surgery, nighttime surgery, postoperative complications

## Abstract

**Background/Objectives**: The objective was to evaluate whether surgical timing (daytime vs. nighttime) influences intraoperative and postoperative outcomes in pediatric supracondylar humerus fractures. **Methods**: A retrospective observational cohort study was conducted at a tertiary center. Pediatric patients aged ≤14 years who underwent surgery for supracondylar humerus fractures between January 2010 and December 2022 were included. Fractures were classified according to the Gartland system. Patients with open fractures, associated neurovascular injury, compartment syndrome, or incomplete follow-up were excluded. Primary outcomes included need for open reduction, reoperation, neuropathy, and loss of joint mobility. Patients were grouped according to the time of surgery: daytime (08:00–22:00) or nighttime (22:00–08:00). Stratified analyses were performed based on fracture severity. **Results**: Eighty-six patients were included: 56 underwent daytime surgery and 30 underwent nighttime surgery. Groups were comparable in age, sex, and fracture severity. Nighttime surgery was associated with a significantly higher rate of open reduction (33.3% vs. 10.7%, *p* = 0.023; RR = 3.11). Reoperation (16.6% vs. 5.4%, *p* = 0.121) and postoperative neuropathy (23.3% vs. 8.9%, *p* = 0.131) were more frequent in the nighttime group, although these differences were not statistically significant. In complex fractures (Gartland III–IV), nighttime surgery was associated with a significantly higher reoperation rate (20.8% vs. 2.6%, *p* = 0.026). **Conclusions**: Nighttime surgery was associated with higher rates of open reduction and reoperation, particularly in complex supracondylar humerus fractures. However, given the retrospective design and limited sample size, these findings may be influenced by residual confounding and should be interpreted as exploratory.

## 1. Introduction

Supracondylar humerus fractures are the second most common fracture in children after distal radius fractures, and the most common in the elbow region. They occur slightly more frequently in boys between the ages 5 and 7. Given their high prevalence, these fractures pose a challenge in daily clinical practice due to the risk of associated complications, including neurovascular injuries, malunion, and loss of full range of joint motion, among others [1,2,3,4,5].

Pediatric bone is characterized by a high remodeling capacity due to active periosteum, abundant vascularity, and dynamic cellular signaling involved in bone healing and regeneration [6]. However, despite this biological advantage, the quality of initial fracture reduction remains critical, as remodeling may not fully correct malalignment, particularly in more complex fractures. Therefore, factors that may influence surgical performance—such as timing of intervention—could indirectly affect both reduction quality and subsequent healing outcomes.

The recent literature has explored various aspects of the management of supracondylar fractures, with particular attention to the timing of surgery. Traditionally, it has been recommended that surgery be performed within the first hours after the injury to reduce the risk of complications. However, it is not uncommon for these injuries to occur outside working hours, often requiring surgical intervention at night [7]. This raises the following question: Can performing surgery outside of regular hours affect the clinical outcome? Current studies suggest that surgeries performed at night may be associated with a higher risk of complications, which could be related to greater fatigue in the surgeon, working with a less experienced team, or reduced availability of resources outside of normal hours [8,9,10]. However, the evidence is inconclusive, with some studies finding no significant association between postoperative outcome and the time of surgery [11,12].

This clinical study aims to analyze the possible association between the timing of intervention for supracondylar fractures in children and the rate of intraoperative complications (need for open reduction to achieve satisfactory fracture reduction) and postoperative complications (need for reoperation, subsequent onset of neuropathy, or loss of mobility with incomplete joint balance).

## 2. Materials and Methods

### 2.1. Design and Participants

A retrospective observational study was conducted to analyze the postoperative clinical outcomes and intraoperative and postoperative complications of pediatric patients with supracondylar humerus fractures treated surgically at a single academic university hospital between January 2010 and December 2022. Prior to initiating data collection, the study protocol was approved by the institutional ethics committee, following the standards and principles of the Declaration of Helsinki. Patients were identified through hospital admission records using standardized diagnosis codes for supracondylar fractures (ICD-9 codes 81240–81249 and 81250–81259).

The sample was incidental, consecutively including all patients who met the inclusion criteria during the study period. Pediatric patients aged 14 years or younger with a diagnosis of supracondylar humerus fracture treated surgically were analyzed. To reduce potential bias, data were obtained exclusively from medical records, and only cases with complete documentation and at least three months of postoperative follow-up were included. The intervention group consisted of patients operated on at night, and the comparison group consisted of those operated on during the day.

Primary outcome variables:

1. Open reduction: conversion from closed reduction during surgery.
2. Reoperation: any unplanned subsequent surgery.
3. Postoperative neuropathy: any new neurological deficit identified after surgery, including both transient and persistent cases.
4. Loss of mobility: incomplete elbow range of motion at final follow-up, as documented in clinical records.

Inclusion criteria:

- Age ≤ 14 years.
- Supracondylar humerus fractures requiring surgical management (Gartland types II–IV; corresponding extra-articular distal humerus fracture types 13-A2 and 13-A3 according to the AO/OTA classification).
- Surgery performed at the same academic university hospital between January 2010 and December 2022.

Exclusion criteria:

- Multiple injuries.
- Pre-existing bone disorders.
- Intra-articular fractures (AO/OTA types 13-B and 13-C).
- Complications during initial management requiring urgent or emergency surgery, including open fracture, neurovascular injury, or compartment syndrome. Neurovascular injury was defined as any documentation of asymmetric or absent distal pulses compared with the contralateral limb, suspected vascular compromise, or neurological deficit, including isolated anterior interosseous nerve palsy.
- Postoperative follow-up <3 months or incomplete clinical data.

**Figure 1:**
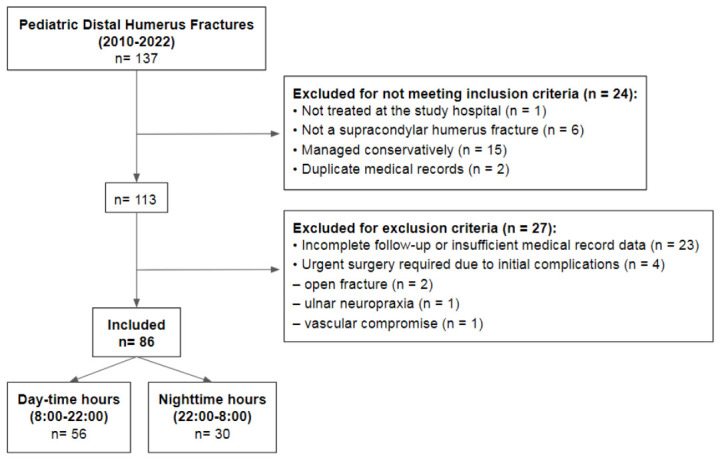
Flowchart of patient selection.

A total of 137 admissions were screened. After applying inclusion and exclusion criteria, 86 patients were included in the final analysis (Figure 1).

### 2.2. Surgical Indications

The decision to operate was made by the on-call attending orthopedic surgeon, with the timing of surgery influenced by logistical and subjective factors. Due to the absence of a dedicated daytime trauma operating room at this institution, patients admitted overnight must either be accommodated within elective surgical lists or treated by the following day’s on-call team. Consequently, there is a tendency to perform children’s surgery urgently—often during nighttime hours—regardless of the specific fracture type or time of admission.

All procedures were performed by board-certified orthopedic surgeons. The pediatric orthopedic team at this institution shares the on-call schedule with the general orthopedic service. Consequently, both daytime and nighttime surgeries were performed by a mixed group of attendings. Residents participated as assistants and did not operate autonomously.

### 2.3. Data Collection

Data were collected retrospectively from the electronic medical records. To ensure confidentiality, an anonymized database was created, in which each patient was designated by a numerical code. Medical record review was performed by orthopedic trauma residents under the direct supervision of orthopedic attending surgeons.

The Gartland classification was used to categorize supracondylar fractures. In the AO/OTA classification, these fractures correspond to extra-articular distal humerus fractures, specifically types 13-A2 and 13-A3.

Regarding the division of the intervention schedule, it was decided to use daytime hours (from 8:00 a.m. to 10:00 p.m.) and nighttime hours (from 10:00 p.m. to 8:00 a.m.).

### 2.4. Statistical Analysis

An initial descriptive statistical analysis was performed. Categorical variables were described using frequencies and percentages. Comparisons of proportions were performed using the Chi-square test or Fisher’s exact test, as appropriate according to subgroup size and expected cell frequencies. A *p*-value < 0.05 was considered statistically significant. All statistical analyses were performed using SPSS Statistics for Windows, version 25.0 (IBM Corp., Armonk, NY, USA).

## 3. Results

### 3.1. Demographics

86 patients were included in the study; 56 underwent surgery during daytime hours (8:00 a.m. to 10:00 p.m.) and 30 during nighttime hours (10:00 p.m. to 8:00 a.m.). Both groups were comparable, with no significant differences in mean age, sex, or overall fracture severity (Table 1). According to the Gartland classification, most fractures were type III (70.9%), followed by type II (26.7%) and type IV (2.3%). Complex fractures (Gartland III and IV) accounted for 69.6% of daytime cases and 80% of nighttime cases.

### 3.2. Variables Analyzed

Statistical analysis (Table 2) identified a higher risk of requiring open reduction to achieve satisfactory reduction in patients operated at night (33.3% vs. 10.71%, *p* = 0.023). The relative risk indicates that in these patients, the risk of requiring open reduction is three times higher than during the day (RR = 3.11), with this risk decreasing by 22.59% if patients underwent surgery during the day.

Regarding the need for reoperation, 16.6% of patients undergoing nighttime surgery required a second procedure, compared to 5.36% of those undergoing daytime surgery (*p* = 0.121). The relative risk was 3.09, indicating more than triple the probability of reoperation in the nighttime group, with an absolute risk reduction of 11.24% in the case of daytime surgery. Reoperations were mainly due to unsatisfactory initial reduction (2 patients operated at daytime vs. 3 in nighttime), followed by surgical revision for postoperative ulnar nerve injury (1 in daytime vs. 2 in nighttime).

The incidence of postoperative neuropathy was higher in surgeries performed at night (23.3% vs. 8.93%, *p* = 0.131). The relative risk was 2.61, indicating that neuropathy was more than twice as likely to occur in the nighttime group.

Postoperative loss of mobility occurred in 16.1% of daytime surgeries and 16.6% of nighttime surgeries (*p* = 1.000), indicating no difference between groups.

### 3.3. Stratified Analysis by Fracture Severity According to the Gartland Classification

The distribution of fracture severity was comparable between groups. However, complex fractures were slightly more frequent in the night group (Gartland III 73.3% vs. 69.6%, *p* = 0.912; Gartland IV 6.6% vs. 0%, *p* = 0.119). Therefore, a stratified analysis by fracture severity was performed (Table 3) to account for this variable and minimize its potential confounding effect.

In the subgroup of patients with Gartland II fractures, low frequencies were found for all perioperative complications studied. A higher proportion of postoperative neuropathy was observed in the nighttime group (33.3% vs. 5.88%; *p* = 0.155). These findings suggest that in low-complex fractures, the chosen time of surgery may have less impact on postoperative outcomes.

In contrast, larger differences were observed in patients with complex fractures (Gartland III and IV). The need for open reduction was greater in the nighttime group (37.5% vs. 15.38%; *p* = 0.090), with an absolute risk reduction of 22.12% if surgery was performed during the day. The reoperation rate was greater at night (20.8% vs. 2.6%; *p* = 0.026), indicating a relative risk of up to eight times higher probability of reoperation in these patients. Postoperative neuropathy was slightly higher in the nighttime group (20.83% vs. 10.3%; *p* = 0.283).

These findings suggest a potential association between surgical timing and complication rates, particularly in complex cases, although causality or independence from other factors cannot be established based on the present study design.

## 4. Discussion

After analyzing the impact of surgical timing on the management and postoperative evolution of pediatric patients with supracondylar fractures, the results show a higher proportion of complications in surgeries performed at night.

The Gartland classification was used because it is widely applied in routine clinical practice, with most physicians familiar with its use. Furthermore, it has demonstrated high interobserver reliability and provides guidance for surgical management [13,14,15]. Regarding the division of the intervention schedule, time intervals were defined to reflect the potential impact of nighttime working conditions on surgical performance. Night shifts are associated with increased fatigue, higher workload, reduced concentration, and limited resource availability; however, these factors were not directly measured. At our institution, on-call shifts last 24 h, meaning that nighttime procedures are typically performed after a full working day.

In this study, nighttime surgery was associated with a greater need for open reduction, as well as a greater need for subsequent reoperation, which was more evident in the subgroup of complex fractures (Gartland III and IV). Although not statistically significant, complex fractures were more frequent in the nighttime group, representing a potential confounding factor.

In the sample studied, a higher risk of requiring open reduction was found in night-time procedures (33.3% vs. 10.71%, *p* = 0.023). Although considered an intraoperative complication, open reduction may also reflect fracture complexity and the need to achieve adequate alignment when closed reduction is not feasible. Therefore, this outcome may be influenced by baseline fracture characteristics in addition to intraoperative factors. Previous studies have indicated that, whenever technically possible, closed reduction should be prioritized, as it is associated with lower morbidity, lower risk of joint stiffness, and shorter hospital stay. Barik et al. (2023) [16] conducted a meta-analysis comparing the aesthetic and functional outcomes of patients undergoing open and closed reduction. They concluded that the functional results were superior in the group treated with closed reduction (98.5% of patients achieved a satisfactory functional result, compared to 93.4% in the open reduction group). Lin-Guo et al. (2018) [17] conducted a systematic review and meta-analysis, finding no significant differences between the two techniques in terms of functional and aesthetic results, ulnar nerve injury, or infection rate. Similarly, Hussein al-Algawy et al. (2019) [18], in a retrospective study, despite also finding no significant differences in functional outcomes or complications, did find that it can be associated with a shorter hospital stay. Moreover, Alshaynawi et al. (2022) [19] conducted a systematic review, in which they observed a higher rate of reoperation in the group of patients treated by open reduction due to a higher incidence of heterotopic ossification in cases treated with this technique.

The association between nighttime surgery and open reduction is likely multifactorial. A higher proportion of complex fractures in the nighttime group may partially explain this finding. In addition, factors related to the surgical environment—such as fatigue, team dynamics, and resource limitations—may contribute. However, these variables were not directly assessed in the present study and should therefore be interpreted as potential explanatory hypotheses rather than confirmed mechanisms. Alternative explanations, including case selection bias, institutional workflow, and variability in surgeon decision-making, should also be considered.

Reoperation rates were higher in complex fractures treated at night, mainly due to unsatisfactory reduction. Taking into account the study’s limitations, these findings suggest that, whenever clinically appropriate, complex fractures may benefit from prioritization for daytime surgery, even if this implies a short delay.

No statistically significant differences were observed regarding the incidence of postoperative neuropathy or final joint balance. Loss of mobility was defined based on clinical documentation of incomplete range of motion, without a standardized quantitative threshold, which may limit the assessment of its clinical significance. In our study, postoperative neuropathy included both transient and persistent deficits, which may overestimate its clinical relevance, as many of these are temporary and resolve spontaneously. Only a small proportion of cases required surgical revision for ulnar nerve injury. Consistently, Ramachandran et al. (2006) [20], in their retrospective study of 37 neuropathies associated with supracondylar fractures, found that 62% were iatrogenic, and 75% of these cases recovered completely without surgical intervention.

Continuing the review of the literature along these lines, Albrahim et al. (2023) [21], in a retrospective study, analyzed the difference in complications by dividing the surgical schedule into “working hours” (from 8:00 a.m. to 3:00 p.m.) and “non-working hours” (3:00 p.m. to 8:00 a.m.), finding a higher overall complication rate in the second group (5.7% vs. 17.6%; *p* = 0.05). Similarly, Aydoğmuş et al. (2016) [8] classified the schedule as “daytime” (8:00 a.m. to 5:00 p.m.) and “after hours” (5:00 p.m. to 8:00 a.m.), finding a higher rate of suboptimal fixation outside of normal hours (9.1% vs. 36.2%; *p* = 0.005). The group led by Paci et al. (2018) [9] used a similar time subdivision to the previous one: “daytime” (6:00 a.m. to 4:00 p.m.) and “after hours” (4:00 p.m. to 6:00 a.m.); it also included a separate subgroup for “nighttime” (11:00 p.m. to 6:00 a.m.). In the latter, they found a higher incidence of malunion (8% versus 0.9% in the rest of the schedules analyzed, *p* = 0.05). Wendling-Keim et al. (2019) [22] reported a higher proportion of postoperative paresthesias in patients who underwent surgery between 10:00 p.m. and 2:00 a.m. Finally, Buget et al. (2022) [10] in a prospective observational study, compared the “daytime group” (7:30 a.m. to 6:30 p.m.) with the “nighttime group” (6:30 p.m. to 7:30 a.m.); their findings show significantly longer surgical times during the night shifts (84.32 vs. 114.66 min on average in the night group, *p* = 0.0001), as well as a higher overall morbidity rate (2.3% vs. 15.4%).

However, there is no clear consensus in the literature, with some studies finding no association between the time of surgery and postoperative outcomes. Ismayil et al. (2022) [23] conducted a meta-analysis that included 14 studies, with a total sample of 1263 patients. The included studies stratified fractures according to the time elapsed since the injury, using different subdivisions, with the most frequent grouping being <8 h from injury versus >8 h to surgery. Although no statistically significant differences were observed in the need for open reduction—the main variable of the study—they interpreted this finding as support for deferring surgeries that are admitted at night and performing them during the day, given that the delay in surgery does not translate into a higher complication rate. Balakumar et al. (2012) [24] conducted a retrospective study with 77 patients, dividing the sample into patients operated on during the “day” vs. “night” (the authors do not specify the time subdivision used). In their case, they found no differences in the main variable analyzed, loss of reduction (19% vs. 18%, *p* = 1.00). Similarly, the studies by Okkaoglu et al. (2021) [25] and Tuomilehto et al. (2018) [26] found no differences in the quality of reduction or fixation, respectively, in patients operated on at night.

These discrepancies may be explained by differences in study design, definitions of surgical timing, fracture severity, and institutional organization. Variability in surgeon experience, resource availability, and outcome definitions further limits direct comparison. Therefore, the impact of surgical timing is likely multifactorial and context-dependent.

This study has several limitations. Its retrospective design and relatively small sample size may limit statistical power and increase the risk of type II error. This may explain the presence of clinically relevant differences that did not reach statistical significance. Therefore, these findings should be interpreted as trends rather than definitive associations.

An important limitation is the lack of data regarding time from injury to surgery, as this variable was not consistently documented across medical records. This is particularly relevant, as surgical delay may influence fracture reduction difficulty and postoperative outcomes. In addition, several other potentially relevant variables were not available, including surgeon-specific experience, operative duration, and prior workload. The absence of these factors further limits the ability to fully account for confounding variables. Outcome definitions such as postoperative neuropathy and loss of mobility were based on clinical documentation, which may limit the precision of outcome characterization. Reoperation was analyzed as a composite outcome including different clinical indications, such as malreduction and nerve-related complications; due to the limited number of events, separate analysis of these subgroups was not feasible, which may limit the interpretation.

Furthermore, no multivariable regression analysis was performed. Given the limited number of events for several outcomes, such analysis could have resulted in overfitting and unstable estimates. Therefore, the absence of multivariable adjustment should be considered when interpreting the observed associations, as residual confounding cannot be excluded. In addition, multiple comparisons were performed without formal adjustment, which may increase the risk of type I error. These findings should therefore be interpreted with caution.

It is also important to note that the definition of surgical timing used in this study (08:00–22:00 vs. 22:00–08:00) differs from that applied in other reports, where “after-hours” surgery is sometimes defined as beginning in the mid-afternoon. This discrepancy may limit direct comparability between studies. Our time classification was designed to particularly reflect the 24-h on-call system, in which nighttime procedures are typically performed after a full working day. However, this approach may not be directly generalizable to other healthcare settings. In addition, the definition of nighttime encompassed a broad time interval (22:00–08:00). Differences between early and late nighttime procedures were not analyzed, and potential fatigue-related effects may have been diluted by this grouping.

Another possible limitation is that the minimum follow-up period of 3 months may be insufficient to fully capture long-term outcomes such as malunion, complete functional recovery, or late nerve recovery. This may result in underestimation of certain complications that manifest beyond the early postoperative period. Furthermore, radiographic alignment at healing was not analyzed due to heterogeneous postoperative imaging protocols, which could have introduced bias. Given that minor angular deviations may not correlate with functional impairment, our analysis focused on clinical outcomes. However, the absence of standardized radiographic parameters may limit the ability to further characterize the underlying mechanisms of outcomes such as malreduction and subsequent reoperation.

Finally, as a single-center study, these findings may not be broadly generalizable, as they reflect the specific surgical practices and organizational structure of our institution. In particular, the absence of a dedicated trauma operating room and the characteristics of our on-call system may have influenced surgical timing decisions, and these results may differ in centers with more structured trauma systems or dedicated pediatric orthopedic teams.

Despite these limitations, this study provides real-world clinical data on the potential influence of surgical timing on intraoperative and postoperative outcomes in pediatric supracondylar fractures. Although causality cannot be established, the findings suggest that, whenever clinically appropriate, complex fractures (Gartland III and IV) may benefit from prioritization for daytime surgery.

## 5. Conclusions

Although our findings suggest a trend toward increased open reduction and reintervention rates in pediatric supracondylar fractures treated at night, these results should be interpreted with caution. By providing data from actual clinical practice, this study contributes to the current body of evidence; however, its exploratory nature—stemming from a retrospective design and a limited sample size—highlights the need for further research. These preliminary findings should therefore serve as a basis for larger, adequately powered prospective studies to better account for potential confounding factors.

## Figures and Tables

**Table 1:** Demographic characteristics of the population.

	Total	Daytime Hours	Nighttime Hours	*p*-Value
N	86	56	30	
Mean age	5.57	5.71	5.3	0.225
Women	40	24/56 (42.9%)	16/30 (53.3%)	0.911
Men	46	32/56 (57.14%)	14/30 (46.6%)	0.911
Gartland II	23	17/56 (30.35%)	6/30 (20%)	0.436
Gartland III	61	39/56 (69.64%)	22/30 (73.3%)	0.912
Gartland IV	2	0/56 (0%)	2/30 (6.6%)	0.119

**Table 2:** Intraoperative and postoperative complications.

	Daytime Hours	Nighttime Hours	*p*-Value	RR	ARR (%)
Open reduction	6/56 (10.71%)	10/30 (33.3%)	0.023 *	3.11	22.59%
Reoperation	3/56 (5.36%) 2 Malreduction 1 Ulnar nerve revision	5/30 (16.6%)	0.121	3.09	11.24%
Neuropathy	5/56 (8.93%)	7/30 (23.3%)	0.131	2.61	14.37%
Loss of mobility	9/56 (16.1%)	5/30 (16.6%)	1.000	1.03	0.5%

* Statistically significant *p*-value. RR: relative risk. ARR: absolute risk reduction, expressed as percentage.

**Table 3:** Intraoperative and postoperative complications, after performing stratified analysis according to fracture severity.

Gartland	Variable	Daytime Hours	Nighttime Hours	*p*-Value	RR	ARR (%)
II	Open reduction	0/17 (0%)	1/6 (16.6%)	-	- **	- **
	Reoperation	2/17 (11.76%)	0/6 (0%)	-	- **	- **
	Neuropathy	1/17 (5.88%)	2/6 (33.3%)	0.155	5.66	27.42%
III–IV	Open reduction	6/39 (15.38%)	9/24 (37.5%)	0.090	2.44	22.12%
	Reoperation	1/39 (2.56%)	5/24 (20.83%)	0.026 *	8.13	18.27%
	Neuropathy	4/39 (10.3%)	5/24 (20.83%)	0.283	2.02	10.53%

* Statistically significant *p*-value. RR: relative risk. ARR: absolute risk reduction, expressed as percentage. ** RR and ARR were not calculated in some subgroups due to small sample size and zero-event cells.

## Data Availability

The data presented in this study are available from the corresponding author upon reasonable request. Due to ethical restrictions regarding patient privacy, the data cannot be made publicly available.
